# Inclusive pedagogy in online simulation‐based learning in undergraduate nursing education: A scoping review

**DOI:** 10.1111/jan.16284

**Published:** 2024-06-28

**Authors:** Lisa Langan, Kate Frazer, Andrew Darley, Lizbeth Goodman, Freda Browne, Patrick Fulfilled, Phil Halligan, Catherine Redmond

**Affiliations:** ^1^ MTU Department of Nursing and Healthcare Sciences Munster Technological University Kerry Ireland; ^2^ UCD School of Nursing, Midwifery and Health Systems University College Dublin Dublin Ireland; ^3^ UCD School of Mechanical and Materials Engineering University College Dublin Dublin Ireland

**Keywords:** inclusive pedagogy, nurse educator, online simulation‐based learning, student diversity, undergraduate nursing student

## Abstract

**Background:**

Equality, diversity and inclusion initiatives seek to embed the concept of inclusive pedagogy to promote inclusive educational environments. However, no evidence synthesis exists which examines whether and how the concept of inclusive pedagogy is addressed in online simulation‐based learning in the undergraduate nursing education literature.

**Aims:**

To map the evidence regarding the adoption of inclusive pedagogy in online simulation‐based learning in undergraduate nursing education.

**Design:**

A scoping review.

**Methods:**

Data were extracted, synthesized and presented in narrative and table format.

**Data Sources:**

A systematic search of five databases and five sources of grey literature was conducted to search literature published between 1st January 2010 to 1st June 2022.

**Results:**

Thirty‐eight papers published between 2011 and 2022 were included. The results are presented under three identified themes: (1) Learner diversity; (2) Theoretical frameworks promoting equality, diversity and inclusion in online simulation and (3) Online simulation feedback.

**Conclusion:**

Inclusive pedagogy has not been considered or embedded in its entirety in online simulation in undergraduate nursing education literature.

**Implications for the profession and/or patient care:**

Utilizing an inclusive pedagogy framework may prove advantageous in generating inclusive teaching approaches to support all students.

**Impact:**

This review will interest educators and managers that wish to incorporate equality, diversity and inclusion initiatives in nursing education.

**Reporting Method:**

This scoping review has adhered to the EQUATOR guidelines: the Preferred Reporting Items for Systematic Reviews and Meta‐Analysis extension for Scoping Reviews checklist.

**Patient or Public Contribution:**

No Patient or Public Contribution.

## 1 INTRODUCTION

Student rights and inclusion in education regardless of diversity are internationally recognized (UN, 2006; UNESCO, 2020). As yet, no universal definition of inclusion within education exists. It can be conceptualized depending on various positions as it is considered highly context and philosophically bound (Messiou, 2017; Nilholm, 2021). Equally, the term diversity is difficult to define. It is seen as a multi‐dimensional concept that is dependent on cultural context and level of awareness of difference (Claeys‐Kulik et al., 2019). Leaders in education have been influenced by the UN Salamanca Statement (UNESCO, 1994), which emphasizes pedagogy, learner‐centredness, embracing diversity and the inclusion of all (Carballo et al., 2021; Floretta, 2021). Thus, a plethora of equality, diversity and inclusion (EDI) initiatives have been developed (Claeys‐Kulik et al., 2019; EUA, 2021) leading to more inclusive approaches in education and to the emergence of the concept of inclusive pedagogy.

## 2 INCLUSIVE PEDAGOGY IN HIGHER EDUCATION

According to Florian and Spratt (2013, p.119), inclusive pedagogy refers to ‘an approach to teaching and learning (T&L) that supports teachers to respond to individual differences between learners, but avoids the marginalisation that can occur when some students are treated differently’. Many authors have developed inclusive pedagogy frameworks (Florian & Black‐Hawkins, 2011; Florian & Spratt, 2013; Livingston‐Galloway & Robinson‐Neal, 2021; Moriña, 2021). Other EDI instructional design frameworks including Inclusive Design, and Universal Design for Learning (UDL) also promote inclusion. Inclusive design originates from digital technologies and adapts a ‘one size fits one’ approach (Goto, 2019, p. 31), and ‘considers the full range of human diversity concerning ability, language, culture, gender, age and other forms of human difference’ (Inclusive Design Research Centre (IDRC), 2023, p. 1). Universal Design for Learning (UDL) originates from the Center for Applied Special Technology (CAST) and is based on universal design (UD) theory with the aim to include all students (Rogers‐Shaw et al., 2018; Dalton et al., 2019).

Moriña's (2021) framework on inclusive pedagogy draws heavily from previous research evidenced in a systematic review. This framework is particularly relevant in higher education as it encourages educators to reflect on their underlying (1) beliefs, (2) knowledge, (3) design and (4) actions of EDI approaches to T&L (Moriña, 2021). The framework is rooted in the belief that students are valuable contributors to the T&L process and are seen as an asset to the T&L environment (Gale & Mills, 2013). This shifts the focus from providing learning opportunities for most students to focusing on providing opportunities for all students (Florian & Black‐Hawkins, 2011). To achieve this, educators need to have an in‐depth understanding of how and what students learn, and what students' individual needs are including disabilities (Rouse, 2009). In adopting an inclusive pedagogical approach, educators not only encourage difference, they celebrate it, and see difference as a strength (Florian & Spratt, 2013). This approach creates the optimal environment for the inclusion of all individuals through the intentional adoption of repeatable, flexible and individualized learning in an inclusive learning environment. Thus, inclusive pedagogy supports not only learner diversity but also the diversity in which students learn (Gale & Mills, 2013).

## 3 INCLUSIVE PEDAGOGY IN ONLINE NURSING SIMULATION

Nurse educators have responded to the call to be inclusive in T&L approaches (Davis et al., 2022). This has resulted in new online T&L strategies, for example, online simulation‐based learning (SBL). Simulation is a highly effective T&L approach both in person (Piot et al., 2022; Vandyk et al., 2018), and in the online environment (Padilha et al., 2019; Shorey & Ng, 2021; Tolarba, 2021; Choi et al., 2022; Cant et al., 2023). Online simulation approaches broadly foster inclusion by increasing accessibility and alternative mediums that allow students to repeat complex scenarios (Cobbett & Snelgrove‐Clarke, 2016; Hunn, 2018; Díaz‐Guio et al., 2021;). These approaches provide students with the opportunity to assess their own learning, ask questions, and receive feedback from educators (Cowperthwait et al., 2021). Online simulation also provides an inclusive learning environment where students have the space to share their experiences with one another and provide students within a platform to enhance their teamwork (Cowperthwait et al., 2021; Diaz & Walsh, 2021), closed loop communication (Diaz & Walsh, 2021) and collaborative skills with interprofessional and diverse communities (Wright et al., 2021).

Online simulation approaches have the potential to advance inclusion of all in other ways through the adoption of variation in skin tone and diverse standardized patients (Foronda et al., 2022). Yet there is also evidence that online simulation may still lack diversity in patient characteristics and conditions (Conigliaro et al., 2020; Craig et al., 2022), with many commercial recorded simulations featuring young, pale, well‐built actors (Conigliaro et al., 2020). In nursing education, the impact is significant as the lack of diverse representation limits the discussions between educators and students about issues such as diverse patients, stigma, stereotyping and subtle biases, and may act to enforce implicit biases (Stone et al., 2020). Studies have shown that implicit bias contributes to health care disparities and lower quality patient care (Chapman et al., 2013; Narayan, 2019) and therefore must be a key component in strategies of simulation design (Waxman et al., 2022). In addition, the characteristics and diversity of the learner group must equally be taken into account and provide evidence‐based components of cultural diversity within the simulation design and scenarios (INACSL, 2021e), which supports the equity, diversity, and inclusivity of all involved in the simulation (INACSL, 2021d).

A number of nurse researchers have taken different approaches to integrating EDI in face‐to‐face simulation programmes through the inclusion of diverse patient populations (Buchanan & O'Connor, 2020), through co‐designing with local communities (Holz & May, 2023; Ibrahim et al., 2023; Nakajima et al., 2022) and service users (Mitchell & Leontino, 2023; Ozkara San et al., 2023). Further EDI initiatives are evidenced through the development of EDI checklists for clinical simulation (Craig et al., 2022) inclusive T&L strategies (Egilsdottir et al., 2021) and inclusive learning environments (Carmody et al., 2020; Vaughn et al., 2022). It is unknown to what extent is EDI represented and integrated in online nursing simulation literature. In this scoping review, we ask whether and how the concept of inclusive pedagogy is addressed in online simulation‐based learning in the undergraduate nursing education literature. For the purpose of this review, inclusive pedagogy is defined as ‘an approach to teaching and learning that supports teachers to respond to individual differences between learners, but avoids the marginalisation that can occur when some students are treated differently’ (Florian & Spratt, 2013: p. 119).

## 4 THE REVIEW

### 4.1 Aim

This review aimed to identify whether and how the concept of inclusive pedagogy is addressed in online simulation‐based learning in the undergraduate nursing education literature. Adopting a scoping review methodology, this paper aims to answer the following research question:

### 4.2 The objectives of the scoping review were:

*How is inclusive pedagogy evidenced in online simulation‐based learning in the undergraduate nursing education literature?*

- To systematically identify and map the evidence to date regarding the use of inclusive pedagogy in online simulation‐based learning in undergraduate nursing education.
- To identify the enablers and barriers of inclusive pedagogy in online simulation‐based learning in undergraduate nursing education.
- To explore nursing students‘ perceptions and experience of inclusive pedagogy and its use in online simulation‐based learning.
- To explore the beliefs, theoretical frameworks, knowledge and practice that underpin nurse educators‘ conceptualisations and development of inclusive pedagogy in online simulation‐based learning.

## 5 METHODS

### 5.1 Design

This scoping review was informed by the best guidance on scoping reviews (Arksey & O'Malley, 2005; Bradbury‐Jones et al., 2022; Levac et al., 2010; Peters et al., 2020; Pollock et al., 2020, 2023) and follows the guidance of the PRISMA ScR Checklist (Tricco et al., 2018) which is detailed in Supporting Information file 1 (Langan et al., 2022a) and the published protocol for this review (Langan et al., 2022b).

### 5.2 Search methods

The Joanna Briggs Institute's (JBI) three‐step search strategy on scoping reviews guided this scoping review which included an initial search, a full search and a manual screening of reference lists of included studies for review by the author team (Peters et al., 2020). The PCC mnemonic (Population, Concept and Context) was used (Peters et al., 2020). In this instance, the population identified are undergraduate nursing students and nurse educators; the concept is inclusive pedagogy, and the context is online simulation‐based learning in undergraduate nursing education. For the concept of inclusive pedagogy search terms included Moriña's (2021) four components: (1) Beliefs, (2) Knowledge, (3) Design and (4) Actions. Synonyms for these four concepts including additional relevant keywords and search terms from other papers on inclusive pedagogy were added to search strategies for each database as they arose, and were documented accordingly (Page et al., 2021). Ongoing review of the search terms was undertaken by the research team and an expert university‐based research librarian, as guided by best practice guidelines in conducting scoping reviews (Pollock et al., 2020). The final search terms utilized in the review can be seen in Table 1.

**TABLE 1:** PCC and Search Strategy.

PCC	Search strategy
Population/Participants‐Undergraduate nursing students involved in online SBL innursing education and nursing educators of undergraduate nursing students	(educator* OR instructor* OR lecturer* OR teacher* OR tutor* OR professor* OR trainer* OR preceptor* OR academic* OR coach* OR undergraduate* OR freshman OR freshmen OR sophomore* OR undergrad* OR underclassman OR upperclassman OR student* OR ‘co‐ed’ OR novice* OR trainee* OR participant* OR scholar* OR learner* OR pre‐reg* OR ‘pre licensure’ OR baccalaureate OR fresher* OR tutee) AND(nurs*)
Concept: Inclusive pedagogy	((‘inclusive pedagogy’ OR value OR merit* OR worth OR principle* OR ethic* OR moral* OR standard* OR belief* OR acceptance OR bias OR assumption OR attitude* OR confidence OR expectation* OR judgement* OR opinion* OR theory OR thinking OR respect OR understanding* OR hypothesis OR impression* OR intuition OR presumption* OR knowledge OR abilit* OR expertise OR insight* OR philosophy OR proficiency OR comprehension OR action OR activity OR process OR steps OR practice OR EDI OR equality OR equity OR diversity) AND (inclus*)) OR ((inclusive OR universal OR participat* OR good OR UX OR UDI OR UDL OR ‘design for all’ OR ‘user experience’ OR ‘codesign’ OR ‘co‐design’ OR adaptability OR flexibility OR learner‐cent* OR ‘active participation’) AND (design))
Context: Online SBL in undergraduate nursing education	((online OR internet OR web OR cyber* OR ‘distance learning’ OR ‘blended learning’ OR video OR hybrid OR telepresence OR digital OR virtual OR augmented OR computer OR 3D OR gamification OR ‘serious gam*’) AND (simulat* OR ‘simulation based education’ OR ‘SBE’)) AND (learning OR education* OR scholarship OR study OR teaching OR training OR pedagogy OR tuition OR instruction OR coaching OR practise OR practice OR curriculum OR curricula OR ‘problem based learning’ OR university)
Strings 1, 2 and 3 were combined to identify articles that will undergo title and abstract screening	

The databases searched were: Applied Science and Technology Full Text (H.W. Wilson/EBSCO) (Computer Science); Cumulative Index to Nursing and Allied Health Literature (CINAHL) (EBSCO) (Nursing); ERIC (Educational Resources Information Center) International (Proquest) (Higher Education); Pubmed (Nursing) and APA PsycInfo (Nursing).

### 5.3 Inclusion and exclusion criteria

The selection of studies for review strictly followed the inclusion and exclusion criteria as seen in Table 2.

**TABLE 2:** Inclusion and exclusion criteria.

Inclusion criteria	Exclusion criteria	Rationale
Studies on inclusive pedagogy in the context of online SBL in undergraduate nursing education.	Studies on inclusive pedagogy in any other context of nursing education including postgraduate nursing education. Studies outside of nursing education.	Aligns with the research question and corresponding aims and objectives. Only studies related to the PCC will be included and therefore will aim to answer the research question.
Studies on online SBL in undergraduate nursing education.	Studies on in‐person SBL in undergraduate nursing education.	Online SBL is the context of this review.
All primary peer‐reviewed papers will be included.	Text and opinion papers will be excluded.	The inclusion of peer‐reviewed papers in this topic will ensure the quality of published work.
Grey literature sources to include exploratory studies, discussion papers, conference proceedings and graduate theses/dissertations will be included.	Any other type of paper will be excluded.	To capture a broad overview of the research topic and in keeping with a realistic timeframe.
The literature search will be limited to between 1st January 2010 to 1st June 2022.	Any papers published before 1st January 2010.	Online simulation in nursing education only started to emerge in the early 2000s, with significant technological advancements over the last 10 years. Therefore, the period for this scoping review will be from 2010 to 2022.
Papers written in or translated into English language only.	Papers written in a language other than English.	The search will be limited to the English language only due to limited resources for translation.

Both published and unpublished literature were sourced from databases and carefully selected websites. Exploratory studies, discussion papers, conference proceedings, and graduate theses/dissertations were included in the grey literature searches. Grey literature sources include Google; Open Grey; the Inclusive Design Research Centre; the International Nursing Association for Clinical Simulation and Learning (INACSL); and the Simulation Innovation Resource Center (SIRC). The search period was restricted from 1st January 2010 to 1st June 2022, as online simulation in nursing education began to emerge in practice and research in the early 2000s.

### 5.4 Data abstraction

**FIGURE 1:**
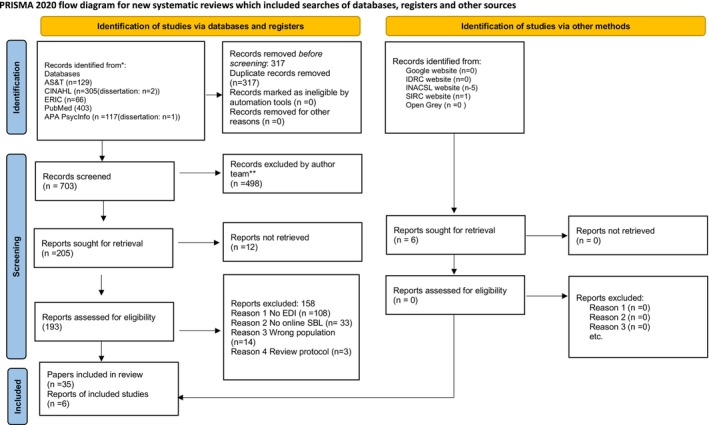
PRISMA‐ScR flow diagram.

The result of the screening process was guided by best practice regarding scoping reviews (Page et al., 2021; Tricco et al., 2018), and is portrayed in the PRISMA‐ScR Flow Diagram (Figure 1). A search of five databases yielded 1020 papers. Following deduplication, 703 papers were uploaded to the Covidence platform where they underwent title and abstract screening. Two hundred and three papers remained for full‐text screening. The full text of 12 papers was not obtainable, therefore 190 papers were eligible for inclusion for full‐text review.

## 6 RESULTS

A total of 32 papers met the eligibility criteria from the selected databases. Some articles reported on the same study from Ireland (Saab et al., 2021, 2022) and Canada (Verkuyl et al., 2016, 2018, 2022a), bringing the total number of primary studies to 29. Geographically studies predominantly came from the USA (*n* = 15), followed by Australia (*n* = 4), Canada (*n* = 1); Korea (*n* = 3); Norway (*n* = 3); Spain (*n* = 2); China (*n* = 1); Ireland (*n* = 1); New Zealand (*n* = 1) and Taiwan (*n* = 1). One study provided evidence across three countries: USA, New Zealand and Australia (Lasater et al., 2019). The settings for the included studies are reported in Table 3.

**TABLE 3:** Country of origin of published studies.

Country of origin of published study	Year of publication											
	2011	2012	2013	2014	2015	2016	2017	2018	2019	2020	2021	2022
USA	1	2		1		2		3	1	2	2	
Canada						1		1				1
China											3	1
Korea												
Australia					1	1			1	1		
New Zealand									1			
Norway											2	1
Ireland											1	1
Spain										1	1	
Taiwan											1	

Grey literature sources yielded six additional sources, from the International Nursing Association for Clinical Simulation and Learning (INACSL) website (*n* = 5), and the Simulation Innovation Resource Center (SIRC) website (*n* = 1). Sources of grey literature all originated from the United States. The final data extraction table in Microsoft Excel was developed, piloted and finalized for extracting data by the author team, detailed in Data S2.

The findings included a variety of methodological approaches including nine quantitative (descriptive cross‐sectional; quasi‐experimental; longitudinal and causal comparative designs), twelve qualitative (two papers from one study (Saab et al., 2021, 2022)) (longitudinal observational cohort studies), and eleven mixed method study designs (descriptive and usability studies). It was noted that there were no randomized controlled trials (RCTs) or systematic reviews in the identified papers. The majority of the primary studies were based in university/college settings (*n* = 17), others were organized in virtual/online settings (*n* = 6), simulation centres (*n* = 3), computer laboratories (*n* = 2), or multimedia rooms (*n* = 1). Six sources of grey literature did not include a study setting including five Standards of Best Practice (2021b; 2021c; 2021d; INACSL 2021e) and one toolkit (NLN, 2017). The majority of the included studies were published prominently in the last 4 years of the search (i.e. 2018–2022), indicating that this topic is a recent phenomenon and is gathering momentum in the nursing education literature.

### 6.1 Evidence of inclusive pedagogy

No primary study or review defined, or made explicit reference to the concept of inclusive pedagogy, therefore it could be argued that no study met the research question. Nevertheless, on careful review of the studies by the author team, the themes were developed based on how the studies aligned with the definition of inclusive pedagogy by Florian and Spratt (2013) and evidence of responding to learner difference in online simulation approaches. Some EDI subcomponents were evidenced in the study's aims and objectives, or from a theoretical framework adopted in the simulation design. Others acknowledged the diversity of learners undertaking the simulation and made adaptations to meet learner needs. The majority of studies reviewed made their first mention of an inclusive pedagogical principle late in their paper, commonly located in their discussion or recommendations section.

One source of grey literature—The National League for Nursing (NLN) Diversity and Inclusion Toolkit (2017) devotes a subsection to inclusive pedagogy in their toolkit. Whilst they do not explicitly define inclusive pedagogy, they affirm the need for pedagogical approaches that are theoretically appropriate, equitable, inclusive, and responsive to diverse perspectives. The INACSL Best Practice Standards (2021) were sourced through grey literature searches and echo some of these recommendations.

Due to the different sources of data obtained, inductive extraction and basic descriptive analysis (Pollock et al., 2023) were undertaken to address the research question. An inductive approach (Pollock et al., 2023) to the analysis stage allowed for amendments to the data charting table to be made by the author team. The following three themes were developed and refined: (1) Learner diversity; (2) Theoretical frameworks promoting EDI in online simulation (3) Online simulation feedback.

### 6.2 Learner diversity

T&L approaches that respond to learner difference is the heart of inclusive pedagogical approaches. Yet few studies position diversity among learners as a primary focus. Five studies embedded learner diversity as a main focus in the aims and objectives. In Scott et al. (2021) study, educators identified the need to proactively respond to diverse students’ needs by creating a flexible virtual programme for students. Carmody et al. (2020) aimed to develop a virtual orientation tool to increase access for all students during COVID‐19, recognizing that students from culturally and linguistically diverse (CALD) backgrounds, sole parents and the newly unemployed were finding it difficult to engage in their learning. Lasater et al. (2019) highlighted the impact of demographics on student learning. Ozkara San (2018) embedded diverse student experiences by utilizing diverse standardized patient simulations to support student learning. Du et al. (2022) also used virtual standardized patients to support the different approaches to history‐taking among diverse learners.

Whilst many of the identified studies indicated diversity among learners through the reporting of various demographic variables, these variables were not discussed further. The most commonly reported variables were age, gender and race/ethnicity. Some studies included demographics regarding education and work experiences (Courtney‐Pratt et al., 2015; Forbes et al., 2016; Johnsen et al., 2021; Lasater et al., 2019; Ozkara San, 2018; Yehle, 2011), or learner's experience with computers, virtual reality, or video games (Saab et al., 2021, 2022; Yehle, 2011) or the length of time learners engaged with T&L strategies (Egilsdottir et al., 2022; Saab et al., 2021, 2022; Yehle, 2011). Some studies also reported on equitable considerations including accessibility to computers (Saab et al., 2022), underrepresented student groups (Dudas & Wheeler, 2020), and students' financial aid status (Musgrove, 2016).

Other studies reported learner diversity as an important and unexpected factor in their findings section. Kim, Kang, and De Gagne (2021)) found that students performed not as well and experienced higher levels of anxiety if the language of the virtual simulation was not in their native language. Friedrich et al. (2020) added that differences in students' knowledge, skills, attitudes, and beliefs can lead to different responses to simulation tasks. Donovan et al. (2018) identified learner differences specific to millennial students, and Egilsdottir et al. (2021) found that diverse student experiences were advantageous in the development of a broad range of mobile technology resources. The remaining studies that mention learner diversity do so in brief terms rather than embedding this concept into their study design and methods (Chang & Lai, 2021; McNeill et al., 2012; Saab et al., 2021; Spalla, 2012; Verkuyl et al., 2022a).

Evidence from grey literature includes best practice standards from the International Nursing Association for Clinical Simulation and Learning (INACSL) supports the consideration of learner diversity in simulation design, and iterates the impact of learner diversity on the learning experience (INACSL, 2021b). The planning for learner diversity incorporates the setting of ground rules (2021), prebriefing and practice time (2021a) and providing preparatory material to support students (2021c), requiring training in student needs assessment (INACSL, 2021e) and psychological safety (INACSL, 2021a). The findings show that few studies planned for learner diversity in their aims or objectives, while the majority briefly mention the significance of this concept indicating a lack of response to learner diversity. The next theme considers the theoretical frameworks adopted in the design of the reviewed studies, some of which consider the diversity of the learner.

### 6.3 Theoretical frameworks promoting EDI in online simulation

Whilst no study specifically utilized an EDI theory/framework, the majority of studies indicate that online SBL approaches are underpinned by educational theoretical frameworks and best practice standards, as outlined in Table 4. Twelve studies cite a learning theory; five studies adopted a nursing simulation theory, while 17 studies used an instructional framework to guide the nursing simulation design and six studies showed no evidence of using a theory or framework (Courtney‐Pratt et al., 2015; Egilsdottir et al., 2021; Friedrich et al., 2020; Kim et al., 2021a; Kim et al., 2021b; Saab et al., 2021, 2022).

**TABLE 4:** Theories, Models and Frameworks cited in the Reviewed Studies.

Theories cited in reviewed study	Papers that identified this theory
Experiential Learning Theory (Kolb, 1984; 2014, 2015)	Spalla, 2012; Foronda et al., 2014; Stanley et al., 2018; Carmody et al., 2020; Dang et al., 2021; Johnsen et al., 2021; Rim & Shin, 2021; Scott et al., 2021; Verkuyl et al., 2016, 2018, 2022b.
Jeffries Simulation Framework and Theory (2005; 2012; 2015)	Foronda et al., 2014; Forbes et al., 2016; Ozkara San, 2018; Chang & Lai, 2021; Johnsen et al., 2021.
Adult Learning Theory	McNeill et al., 2012; Spalla, 2012; Donovan et al., 2018.
Social Constructivism (Dewey, 2007; Vygotsky in Wetsch, 1985; Piaget, 1970 and Bruner, 1966)	Yehle, 2011; Carmody et al., 2020
Leininger's (2006) Theory of Culture Care Diversity and Universality	Ozkara San, 2018
Means End Theory (Gutman, 1982)	Verkuyl, 2022
Self‐Efficacy Theory (Bandura, 1977)	Spalla, 2012
Situated Cognition Learning Theory (Brown et al., 1989)	Musgrove, 2016
Social Learning Theory (Bandura, 1971)	Dang et al., 2021
Social Cognitive Theory (Bandura, 2001)	Dang et al., 2021
Theory of Reasoned Action (Ajzen and Fishbein, 1980)	Verkuyl et al., 2016
**Models and Frameworks cited in Reviewed Study**	**Study that identified this model/framework**
International Nursing Association for Clinical Simulation and Learning (INACSL) Standards of Best Practice (INACSL, 2016)	Ozkara San et al., 2018; Dudas & Wheeler, 2020; Jiménez‐Rodríguez et al., 2020; Arrogante et al., 2021; Rim et al., 2021; Scott et al., 2021.
Situated Cognition (Brown et al, 1989)/situated learning Framework (Paige and Daley, 2009)	Musgrove, 2016; Verkuyl et al., 2016.
Appreciation of the Role of Culture in Health Care) (ARC) Model for Nursing Education towards Cultural Competency (Spalla, 2012)	(Spalla, 2012)
Association for Simulated Practice in Healthcare (ASPIH, 2016) Standards, Framework and Guidance	Johnsen et al., 2021
Association of Standardized Patient Educators (ASPE) (Lewis et al., 2017)	Ozkara San, 2018; Jiménez‐Rodríguez et al., 2020; Arrogante et al., 2021
Cultural Competence and Confidence Model (Jeffrey, 2016a)	Ozkara San, 2018
Debriefing and Good Judgement Model (Rudolph et al., 2007).	Dudas & Wheeler, 2020
Debriefing for Meaningful Learning Process (Dreifuerst, 2012)	Foronda et al., 2016
Diffusion of Innovations Model (Rogers, 2003)	McNeill et al., 2012
Eleven Dimensions of Simulation (Gaba, 2007)	Spalla, 2012; Forbes et al., 2016
Functional Health Pattern Framework (Gordon and Mahriner, 1983)	Du et al., 2022
Fundamentals of Care (FoC) Conceptual Framework (Egilsdottir et al., 2022)	Egilsdottir et al., 2022
Guidelines for Coaching Standardized Patients (Lewis et al., 2017; Wallace, 2007)	Ozkara San, 2018
Intercultural Development Inventory (IDI) (Hammer and Bennett, 1998)	Spalla, 2012
Jeffrey's Transcultural Self‐Efficacy Tool (Jeffrey's, 2016b)	Ozkara San, 2018
Medical Research Council Framework (Craig et al., 2013)	Saab et al., 2021
National League for Nursing Core Competencies of Nurse Educators Framework; (NLN, 2005)	McNeill et al., 2012
Psychological Safety (Rudolf and Raemer, 2014)	Jiménez‐Rodríguez et al., 2020; Arrogante et al., 2021
Tanner Model of Clinical Judgement (2006)	Lasater et al., 2019
Technological, Pedagogical and Content Knowledge Framework (TPACK) Framework (Koehler and Mishra, 2009)	Egilsdottir, 2022
Technology Acceptance Model (Davis, 1989; Davis et al., 2006)	Verkuyl et al., 2016; Verkuyl et al., 2018
Virtual Simulation Design Model (Yehle, 2011)	Yehle, 2011

Many of the models and frameworks used in the reviewed studies promote psychological safety and autonomous learning through alternative, flexible, repeatable, simulation designs evidencing a tailored and responsive approach to individualized student needs and preferences. The most widely adopted theory among the reviewed studies is the Experiential Learning Theory (Kolb, 1984), which posits that knowledge is created through the transformation of experience (Dang et al., 2021; Foronda et al., 2014; Johnsen et al., 2021; Rim & Shin, 2021; Scott et al., 2021; Spalla, 2012; Stanley et al., 2018; Verkuyl et al., 2016, 2018, 2022a). This was followed by the NLN Jeffries Simulation framework and theory, which considers learner attributes such as age, gender anxiety, confidence, and preparedness (Chang & Lai, 2021; Forbes et al., 2016; Foronda et al., 2014; Johnsen et al., 2021; Ozkara San, 2018). Other studies focused on transcultural nursing theory (Ozkara San, 2018; Spalla, 2012), culture care diversity and universality theory (Ozkara San, 2018).

The International Nursing Association for Clinical Simulation and Learning (INACSL) Standards of Best Practice were the most widely adopted standards in the reviewed studies. Studies that embedded these standards evidenced a responsive approach to the inclusion of students through acknowledging the diversity among learners, and the adoption of psychological safety, needs assessments and flexible and repeatable learning (Ozkara San, 2018; Dudas & Wheeler, 2020; Jimenez‐Rodriguez et al., 2020; Arrogante et al., 2021; Scott et al., 2021). Other standards that promote EDI include the Association of Standardized Patient Simulators (ASPE) Standards, which supports a safe work environment (Ozkara San, 2018; Jimenez‐Rodriguez et al., 2020; Arrogante et al., 2021) and Gaba's Guidance on Simulation, advocating for repeatability in learning approaches (Spalla, 2012; Forbes et al., 2016). The findings show that many studies responded to learner diversity as this concept was embedded in some theoretical frameworks adopted in the study design. In these studies, learner diversity was an anticipated concept and was planned for accordingly. The next theme shows the magnitude of information shared by individuals based on their use of online simulation approaches.

### 6.4 Online simulation feedback

The identified papers reveal the perceptions of students, educators and experts following engagement with online simulation approaches as an educational pedagogy, the majority of which were gathered retrospectively. As seen in Supporting Information File 2, twenty‐two out of twenty‐nine studies sought feedback from students. One study by Egilsdottir et al. (2021) showed evidence of a responsive approach and involved students from the beginning in a longitudinal participatory co‐design of a suite of learning resources providing various recommendations as the design evolved. Kim et al., (2021a) also supported a participatory learning approach using a virtual reality perioperative patient simulation approach, which resulted in students advocating for the inclusion of the patient experience in nursing curricula.

Studies which explored student perceptions of their experience of online simulation approaches led to substantial and detailed information. Student preference to online simulation approaches was afforded to the provision of clear instruction (Carmody et al., 2020; Egilsdottir et al., 2022), correction (Egilsdottir et al., 2022) and caring feedback (Carmody et al., 2020; Egilsdottir et al., 2022; Scott et al., 2021). Students preferred the opportunity for reflection (Verkuyl et al., 2016; Johnsen et al., 2021; Rim et al., 2021) to engage anonymously (Egilsdottir et al., 2021) without others watching them (Chang & Lai, 2021; Verkuyl et al., 2016) and with minimal technical interference (Verkuyl et al., 2018). Students valued the inclusion of using ‘real’ people (Egilsdottir et al., 2021; Johnsen et al., 2021) and participation of educators in the online SBL approach (Carmody et al., 2020; Dudas & Wheeler, 2020; Egilsdottir et al., 2022). Students described experiencing a learning environment where they felt safe to make mistakes and learn from them (Dudas & Wheeler, 2020; Saab et al., 2021; Verkuyl et al., 2018). Their experience led to high levels of confidence (Donovan et al., 2018; Egilsdottir et al., 2022; Rim & Shin, 2021; Saab et al., 2021) and satisfaction among students (Foronda et al., 2016; Carmody et al., 2020; Jimenez‐Rodriguez et al., 2020; Scott et al., 2021; Saab et al., 2022) and ultimately they felt prepared for clinical practice (Spalla, 2012; Verkuyl et al., 2018; Jimenez‐Rodriguez et al., 2020).

The evidence highlighted how students also experienced technical challenges (Arrogante et al., 2021; Jiménez‐Rodríguez et al., 2020; Musgrove, 2016; Rim & Shin, 2021; Verkuyl et al., 2016), internet issues (Jiménez‐Rodríguez et al., 2020; Verkuyl et al. 2022a; Yehle, 2011) or navigation issues during their engagement with various online simulation approaches (Kim et al., 2021a; Verkuyl et al., 2018, 2022a). Some students reported that online simulation approaches as lacking warmth (Chang & Lai., 2021; Saab et al., 2021), unrealistic (Yehle, 2011; Chang & Lai., 2021), antisocial (Egilsdottir et al., 2022; Saab et al., 2021), unsuitable (Carmody et al., 2020; Saab et al., 2022) or too long (Foronda et al., 2016). Some students noted disadvantages of online simulation approaches reporting that they cause dizziness (Saab et al., 2021; Verkuyl et al., 2018), motion sickness, vertigo or injury (Saab et al., 2021, 2022). Students also noted the potential to create language barriers (Du et al., 2022; Kim et al., 2021a) with no options to ask questions (Saab et al., 2022). Students also highlighted the time (Carmody et al., 2020; Chang & Lai, 2021) and self discipline (Egilsdottir et al., 2021) or specific equipment such as a smartphone required to engage in the online simulation approach (Foronda et al., 2016). Students recommended providing more information about the T&L approach (Verkuyl et al., 2018, 2022a) along with sufficient time to engage in preparatory material (Chang & Lai, 2021; Kim et al., 2021a; Rim & Shin, 2021). Some students and educators recommended increasing the time of the online simulation approach (Verkuyl et al., 2018), and provide more student access to online simulation approaches overall (Egilsdottir et al., 2021, 2022), recommending embedding online simulation throughout curricula (Egilsdottir et al., 2022).

Educators also provided detailed feedback that primarily focused on their experience as an educator in using online simulation approaches. Educators reported challenges including lacking confidence (Stanley et al., 2018) and feeling nervous using online simulation approaches (Verkuyl et al., 2016). They also emphasized the significant training and expertise (Stanley et al., 2018), pedagogical and digital competence (Egilsdottir et al., 2021), in balancing pressurized workloads and time constraints (Scott et al., 2021). Adopting online simulation approaches required motivation and support (Musgrove, 2016) on top of the need to be entertaining online (Egilsdottir et al., 2022), while navigating technical difficulties (Verkuyl et al., 2018), engaging with ‘black screens’ (Egilsdottir et al., 2022) and resolving a lack of student engagement (Carmody et al., 2020). Educational designers, content creators (Carmody et al., 2020), and other experts (Du et al., 2022; Verkuyl et al., 2018) were consulted during the studies that primarily focused on how to fine‐tune navigation options (Verkuyl et al., 2016), provide additional information, and how to navigate the various online simulation approaches (Rim & Shin, 2021; Verkuyl et al., 2016, 2018). These findings regarding feedback on online simulation align with the INACSL Best Practice Standards which indicate that simulations should be designed with experts and learners (INACSL, 2021e) and evaluated by participants, peer clinicians, educators, stakeholders and all involved in the simulation, requiring a more learner‐centred, facilitative approach to the planning of simulation design (INACSL, 2021a).

## 7 DISCUSSION

The purpose of this review was to identify whether and how inclusive pedagogy is addressed in online simulation‐based learning in the undergraduate nursing education literature. The identified studies were analysed under the key themes of learner diversity, inclusion frameworks used and feedback on simulation experience. None of the reviewed studies explicitly identifies the concept of inclusive pedagogy, suggesting that the concept itself is widely unknown in the nursing simulation literature. Yet the findings show evidence of various responses to learner difference in online simulation approaches. Responding to learner diversity was found through embedding learner diversity into T&L approaches, through the adoption of theoretical frameworks that support learner diversity, and through exploring students' diverse experiences in online simulation approaches albeit the vast majority retrospectively. The findings identified the current guidance in inclusive pedagogy available in the nursing education literature. The NLN (2017) Inclusion and Diversity Toolkit provides specific questions for educators wanting to adopt inclusive pedagogy, which promotes the inclusion of student views. Providing opportunities for students to share their views is fundamental to responding to student needs, but must be joined with deliberate action (Messiou, 2019). An initial step in responding to learner difference is to firstly acknowledge its effect on student learning and secondly, to go deeper into what that impact might be in different student cohorts. To support educators to support current students, clear and specific guidance is warranted. The NLN (2017) has identified specific key points applied to nursing that would benefit from further development.

Many of the reviewed studies report demographic variables (i.e., race/ethnicity, gender, and age) yet Bleich et al. (2015) noted that these demographics are too simplistic to measure impact. In contrast, the few studies that put learner diversity as a main focus found that demographics do impact student learning (Carmody et al., 2020; Du et al., 2022). This is supported by the broader literature which identifies that the student experience in education is dependent on various factors including demographics, culture and socio‐economic processes and policy (Ainscow, 2020). Gilmore et al. (2022) also note that diversity within the nursing profession goes beyond demographics and includes other aspects such as identity and ability. It is clear that student learning is impacted by learner differences, identifying that learner differences need to be a key consideration prior to student engagement with teaching and learning strategies.

Earlier reviews found a lack of support of a learning theory in the majority of studies in both face‐to‐face and virtual reality nursing simulations, recommending that future researchers should explore the learning within simulation and the factors that foster or impede student learning (Koskinen et al., 2023; Lavoie et al., 2018). In contrast, this review showed evidence of utilizing various theoretical frameworks in over half of the reviewed studies. Many of the theories and frameworks utilized contain evidence of responding to learner difference. This led to the use of needs assessments, psychological safety protocols and repeatable learning opportunities for students. Repetition was previously highlighted in earlier reviews as an advantage to students, where they had an option to repeat/practice simulations without the risk of harming patients or themselves (Coyne et al., 2018, 2021). This has changed the dynamic of how students engage with learning as it gives students the autonomy to learn at their own pace and to self‐assess their learning in a safe place without the presence of others. The use of best practice guidelines in the reviewed studies guided educators to plan and design an initial needs assessment to identify knowledge, skills, attitudes, and/or behaviours of their learners. Whilst not explicitly identified as meeting diverse learner needs, this is effectively what educators were doing. In addition, the initial setting of ground rules and expectations observed in these studies helped to provide a psychologically safe learning environment which aids the inclusion of all (Buchanan & O'Connor, 2020; Jeffries, 2022; Turner et al., 2023).

From the literature reviewed here, the inclusion of students in the design of online simulation approaches is limited to one participatory co‐design approach (Egilsdottir et al., 2021). These authors argue that as end users of teaching pedagogies, students should be co‐collaborators in the development and evaluation of T&L approaches to ensure an effective user‐centred design. EDI theorists also believe that students represent a valuable source of knowledge and insight for the effective development of all T&L resources and that participatory co‐design effectively fosters negotiation, participation, and inclusion of the voice of the students (Carballo et al., 2021; Moriña, 2021; O'Connor et al., 2021). However, reports of co‐design within the healthcare education context in general are limited. Theobald et al. (2021) co‐designed a postgraduate specialist course with hospital and academic service providers and concluded that co‐design strengthened the nexus between both entities, improving both learning and employability. Other examples of co‐design for educational interventions have demonstrated how co‐designing is responsive to the needs and interests of both students and educators, and clinical practitioners (Hardie et al., 2022 and Redmond et al., 2023).

There is no evidence of public patient involvement (PPI) in any of the reviewed studies, although one study reported student recommendations advocating for the inclusion of the patient experience in nursing curricula (Kim et al., 2021b). EDI literature firmly supports the inclusion of diverse communities as partners, and co‐creating clinical simulations with individuals with lived experience representative of communities which is imperative to understand diverse patient populations (Picketts et al., 2021; O'Connor & Byrne, 2022; Horta Reis Da Silva & Mitchell, 2024).

### 7.1 Implications for practice and future research

Nurse educators play a crucial role in promoting and responding to diversity among student cohorts. However, this review highlights a gap in the nursing education literature regarding the lack of evidence of inclusive pedagogy as a concept in online simulation approaches, which inadvertently may inhibit educators' ability to respond to growing learner diversity. The findings of this review are pivotal to the planning and reform of nursing curricula and will assist educators in the development and implementation of online simulation approaches with inclusive pedagogy as a central focus. A broad inclusive pedagogical approach is warranted that can be easily applied as novel simulation technologies emerge. In future studies, a clear definition of inclusion and diversity, may clarify what facets of inclusion are embedded in their studies, and how educators prioritize learner diversity. Assuming that all students may require support at different times would set the foundation for educators to adopt a more responsive approach including the use of demographic data to guide educators in the development of inclusive T&L strategies. Utilizing an inclusive pedagogy framework may prove advantageous as it begins with educators' underlying beliefs regarding learner difference, that prompts a responsive approach to working with students, generating inclusive teaching approaches to support all students.

The lack of training in inclusive pedagogy (Moriña & Orozco, 2021) and inclusive education (Moriña, 2022) has been previously highlighted in the broader education literature, leaving educators unprepared and unsupported to apply inclusive pedagogical approaches (Rouse, 2009). Educators need the knowledge and skills to address the structural barriers to support all students and require clear measurable standards related to the achievement of diversity (Murray & Noone, 2022). This review supports the call for educator training (Frazer et al., 2021; Charania & Patel, 2022). Further research should position students and other stakeholders as partners in the design of online simulation approaches.

### 7.2 Strengths and limitations

This review has several strengths, such as addressing a critical research synthesis gap, following methodological rigour and guidance on scoping reviews and using a well‐planned search strategy and data extraction methods (Tricco et al., 2018). The findings provide multiple perspectives on the current position of inclusive approaches to online simulation in undergraduate nursing education. Multiple expert researchers were involved throughout the review process. Nevertheless, it is important to note that this scoping review has several limitations. Firstly, the inclusion and exclusion criteria included studies in the English language only. Secondly, despite contacting the authors directly, the author team could not obtain the full text for 12 papers. Additionally, more sources of grey literature could have been included to broaden the search. No evidence of the concept of inclusive pedagogy was evidenced in any of the reviewed studies, and therefore the enablers and barriers of this concept could not be mapped out. This had implications in meeting the aim and objectives and is a limitation of this review.

## 8 CONCLUSION

Inclusive pedagogy is not only an applied approach to EDI in nursing education, it is a comprehensive approach that seeks to respond and adapt to learner diversity. This review has identified a gap in the existing literature regarding how inclusive pedagogy and its subcomponents are inconsistently evidenced in the online SBL in nursing education literature. While interest in learner diversity and its impact on student learning is gathering momentum, the approaches to it are notably inconsistent and appear tokenistic at times. A collaborative and participatory approach to nursing education may be required to address this gap in evidence and educational practice. Adopting inclusive pedagogy in the development and implementation of online simulation approaches is responsive and supportive to learner difference. By embracing diverse perspectives, the development of alternative, flexible, repeatable simulations that promote the inclusion of all students can be more robustly facilitated, which may positively impact the future of our nursing workforce.

## AUTHOR CONTRIBUTIONS

Lisa Langan lisa.langan@ucdconnect.ie Twitter: @LisaLangan9. Roles: Conceptualisation, Investigation, Project Administration, Visualization, Writing – Original Draft Preparation, Writing‐Reviewing and Editing. Kate Frazer kathleen.frazer@ucd.ie Twitter: @KateF224. Roles: Conceptualisation, Investigation, Project Administration, Visualization, Writing – Review & Editing. Andrew Darley andrew.darley@ucd.ie Twitter: @adarleyresearch. Roles: Conceptualisation, Investigation, Project Administration, Visualization, Writing‐Review and Editing.Lizbeth Goodman lizbeth.goodman@ucd.ie. Roles: Conceptualisation, Project Administration, Supervision, Visualization. Catherine Redmond catherine.redmond@ucd.ie. Roles: Conceptualisation, Investigation, Project Administration, Supervision, Visualization, Writing – Review & Editing. Freda Browne freda.browne@ucd.ie Twitter: @Fredabrowne4. Roles: Conceptualisation, Investigation, Project Administration, Supervision, Visualization, Writing – Review & Editing. Patrick Fulfilled patrick.fulfilled@ucdconnect.ie Twitter: @PatrickFulfill4. Roles: Conceptualisation and Investigation. Phil Halligan phil.halligan@ucd.ie. Roles: Conceptualisation and Investigation, Project Administration, Supervision, Visualization.

## FUNDING INFORMATION

The authors declare that no grants supported this work.

## CONFLICT OF INTEREST STATEMENT

All authors declare no conflicts of interest affiliated with this work.

### PEER REVIEW

The peer review history for this article is available at https://www.webofscience.com/api/gateway/wos/peer‐review/10.1111/jan.16284.

## Supporting information


Data S1.

Data S2.

## Data Availability

Data Availability StatementThe Supporting Information files of this article.Supporting Information File 1. The PRISMA ScR Checklist: https://doi.org/10.6084/m9.figshare.21214004.v1Supporting Information File 2. Data Charting Table: https://figshare.com/articles/dataset/Data_Charting_Table_docx/24556648 Data are available under the terms of the Creative Commons by 4.0 (CC BY 4.0).
