# Supplementary material for: Inclusive pedagogy in online simulation‐based learning in undergraduate nursing education: A scoping review
Source: J Adv Nurs. 2024 Jun 28;81(2):591–606. doi: 10.1111/jan.16284 (PMC11729359; doi:10.1111/jan.16284)
Supplement: Supplementary file 1 — Data S1. [file JAN-81-591-s001.docx]

## Data Charting Table

**SUPPORTING INFORMATION 2**

| **Author(s)**  **and Country** | **Source** | **Study Population** | **Sample size** | **Aims and objectives** | **Main Findings on inclusion** | **Evidence Identifying Learner Diversity** | **Discussion** | **Limitations** | **Recommendations** | **Level of inclusion**  **Students** | **Level of inclusion**  **Educators** | **Level of inclusion**  **Experts** |
| --- | --- | --- | --- | --- | --- | --- | --- | --- | --- | --- | --- | --- |
| Arrogante et al., 2021: Spain | Empirical Study | Fourth-year nursing students | 234 | Rapid response to learner needs due to the COVID-19 pandemic | This online approach could still be used to reduce travel and time costs. | Rapid response to learner needs in response to COVID-19 pandemic | The potential impact of anxiety during the clinical simulation was discussed and addressed through the adoption of prebriefing and psychological safety. Learning from mistakes is advantageous; the practical utility of OSCEs; reduces the theory-practice gap; increases preparedness; time appropriate. | Subcomponents of inclusive pedagogy not addressed | Subcomponents of inclusive pedagogy not addressed | None | None | Consultation |
| Carmody et al., 2020:  Australia | Empirical Study | First-year nursing students | 565 | Preparing for work-integrated learning during COVID-19: How a new virtual orientation tool facilitated access for all | Students specifically appreciated the flexible and responsive approaches taken to communication:  The teaching team identified the need to more frequently reach out and to offer students multiple ways of engaging with educators and the subject content.  Students also faced individual challenges, for example cultural and language barriers to online learning access. | Identified evolving student needs and needs specific to CALD backgrounds. | Some students were slow to accept the new T&L approaches including students from culturally and linguistically diverse (CALD) backgrounds, sole parents, carers and the newly unemployed. COVID-19 highlighted the complexities involved in the rapid change to distance learning and the diverse and evolving needs of students.  Inclusive teaching practices underpin effective preparation for WIL, especially in uncertain times, and must be valued more highly. | Subcomponents of inclusive pedagogy not addressed | Subcomponents of inclusive pedagogy not addressed | Evaluation | Reflection | Consultation |
| Chang & Lai, 2021: Taiwan | Empirical Study | Nursing students in the Adult Nursing and Practice course | 60 | Not related to inclusive pedagogy | Stress/pressure-free friendly learning environment.  Students could repeat the practice indefinitely without instructor supervision; saving time and material resources and costs.  Students were in charge of their learning. The virtual reality teaching approach enables unlimited access to risk-free scenarios, thus reducing student anxiety and improving student confidence. | Notes learner diversity.  The trend of diverse learning methods was identified. | The virtual reality teaching approach is a suitable for adaptable teaching strategy and could supplement T&L approaches for nursing skills. | Subcomponents of inclusive pedagogy not addressed | In accordance with the trend of diverse learning methods, educators must adjust their teaching methods to suit their learners. | Evaluation  Experience | None | None |
| Courtney-Pratt et al., 2015:  Australia | Empirical Study | Second-year nursing students | 497 | Reports on the implementation and evaluation of an immersive 3D cultural empathy simulation. | The sim was highly valued; effective, invaluable, realistic, beneficial, and motivating, enhancing their understanding regarding cultural competence. | Identified that the Cultural Simulation Experience Scale requires further testing with diverse cohorts. | Students’ heightened awareness and recognition of the relevance of cultural competence | Subcomponents of inclusive pedagogy not addressed | Subcomponents of inclusive pedagogy not addressed | Evaluation | None | Review |
| Dang et al., 2021:  USA | Empirical Study | Prelicensure, baccalaureate nursing students in their first medical-surgical course | 160 | Not related to inclusive pedagogy | Immersive telepresence technology can be used for larger student groups and promotes engagement and perceived presence. | Not addressed | Using immersive telepresence roles in simulation provides a means of incorporating larger class sizes to attend simulations without having to physically be present in a simulation centre. | Subcomponents of inclusive pedagogy not addressed | Subcomponents of inclusive pedagogy not addressed | Perceptions | None | None |
| Donovan et al., 2018:  USA | Empirical Study | Senior-level nursing students | 82 | To examine nursing students' perceptions and experiences of a preparatory computer-based simulation prior to a simulated lab experience. | Individualised preparedness  Decreased level of anxiety, and increased confidence in the lab. Reinforcement was a positive aspect of the computer-based simulation scenarios. | Individual learning needs: millennial students | The vSim for Nursing program focused the student on individual learning needs and enhanced individual readiness for the simulation lab learning.  Preparatory computer-based simulation programs improved simulation lab experiences by encouraging individualization of student learning. | One cohort of students used the vSim for Nursing. | Subcomponents of inclusive pedagogy not addressed | Evaluation  Experience | None | None |
| Du et al., 2022:  China | Empirical Study | Full-time nursing undergraduates in four-year courses. | 174 | To evaluate the history-taking skills of nursing undergraduates using a virtual standardised patient, and to explore its independent influencing factors. | The history-taking level of the nursing students was significantly associated with ethnicity, previous academic performance, and Supportive Communicative Scale scores. Potential differences in language use may place other ethnic minorities at a disadvantage in oral expression. | Identified difficulties regarding history taking by some ethnic minority student groups in China. | Ethnicity, previous academic performance and supportive communication skills independently influenced the students' history-taking level. | This study acknowledged that it is unable to comprehensively determine the influencing factors of nursing students' history-taking skills. | Future studies should take into account the need for history-taking assessments in different languages and try to develop versions for minority students, helping them to improve their history-taking skills in nursing education | Evaluation | None | Collaboration team |
| Dudas and Wheeler, 2020:  USA | Empirical Study | Enrolled in upper-division nursing courses during the study period. | 24 | Prelicensure baccalaureate nursing students’ experiences with the use of faculty re-enactment videos as a supplement to debrieﬁng after high-ﬁdelity simulation. | They reported feeling ‘‘safe to make a mistake’’ and not feeling ‘‘judged’’ about their performance during the simulation.  T&L approaches need to be adapted to support the learning of Generation Z students; Videos were personalised for students learning. | Not addressed | They found a connection between simulation, the videos, and a ‘‘just culture” which includes collaboration among students, faculty, and leaders.  Faculty acted on student feedback and responded to student needs.  Flexibility and dynamism are seen as “caring” characteristics.  Highlighted the importance of building of relationships between students and faculty. | Subcomponents of inclusive pedagogy not addressed | Subcomponents of inclusive pedagogy not addressed | Experience | None | None |
| Egilsdottir et al., 2021: Norway | Empirical Study | First-year students, second-year students, third-year students. | 20 | To explore and elicit the perspectives of students regarding how a selection of digital learning resources supports B-PAS learning. | The students participated throughout the development of a prototype.  According to the students, the digital learning resources stimulated learning in seven different ways.  The digital learning resources contributed to student learning individually and collectively. | Identified student needs for mobile technology users.  This study explored multiple digital learning resources with high student involvement and identified the different impacts on students’ learning. | The authors acknowledged the contribution of the participating students in the co-design processes and development of the suite of mLearning tools.  Students valued the invitation to collaborate and influence nursing education content.  The role of facilitation in digital simulation also involves creating a safe learning environment.  Students were influencers in the nursing education content. | The students that participated in this study might not necessarily represent the diversity of the university’s student population but rather those motivated and with the ability to participate in extracurricular activities | The use of the suite of mLearning tools should be tested by the entire student population.  The focus of the study was on the experience of the students with mLearning, not the perspectives of the faculty or preceptors, representing another limitation that should be addressed through future research. | Evaluation  Collaboration team | None | None |
| Egilsdottir et al., 2022: Norway | Empirical Study | First-year nursing students enrolled in a three-year bachelor program and program faculty members. | 24 | To explore how an innovative redesign of a clinical course in a time of pandemic supported nursing students in learning the fundamentals of care in their first year. | Caring facilitation contributes to building confidence;  A flexible T&L approach strengthens professional knowledge: students felt it was tailored to their everyday life activities and obligations | Rapid response to learner needs in response to COVID-19 pandemic | Virtual patient scenarios contributed to integrating different types of knowledge and skills | Subcomponents of inclusive pedagogy not addressed | Subcomponents of inclusive pedagogy not addressed | Experience | Experience | None |
| Forbes Bucknall and Hutchinson, 2016:  Australia | Empirical Study | Final-year nursing students | 31 | Not related to inclusive pedagogy | Participants felt they were able to clarify areas of uncertainty ensuring their learning needs were met, which identified factors that enabled/ impeded their ability to manage a deteriorating patient. | Not addressed | The usefulness of the video recordings was inconclusive. | Lack of previous simulation experience.  The positioning of the camera affected the usefulness of recordings. | Modiﬁcation of the video camera glasses, to improve visual-ﬁeld synchronisation with participants' actual visual ﬁeld, is recommended to further explore this technology for enhancing student performance. | Feasibility test | None | None |
| Foronda et al., 2016:  USA | Empirical Study | Accelerated BSc Nursing students. | 54 | The purpose of this study was to report students’ experience with vSim for Nursing. | The simulations resulted in both positive and negative feedback from students  Two students reported they preferred the manikin-based simulation. | Not addressed | Virtual simulation has a wide range of applications and warrants further exploration. | The evaluation survey was anonymous, and no demographic data were collected which limited generalizability.  Students who did not bring a smartphone may not have been able to participate. | Future virtual simulation software designers may consider the ability of users to multitask. | Evaluation  Experience | None | None |
| Foronda et al., 2014:  USA | Empirical Study | Baccalaureate nursing students | 9 | Students engaged with a Virtual clinical simulation. | The simulations resulted in both positive and negative feedback from students  Students expressed having less anxiety, knowing what to expect, and having “better ﬂow” with communication  After the second simulation, students expressed feeling more prepared and that they enjoyed the second simulation more. | Not addressed | Repetition of hearing the ISBAR was highlighted as an advantage to students | One student was unable to participate in this study because she could not log in. | A need for sound log-in directions and troubleshooting solutions.  A trial run to ensure access to the platform and that equipment is working. An information technologist if required. Healthcare professionals could play the role in simulations for interprofessional education. | Evaluation | None | None |
| Friedrich et al., 2020:  USA | Empirical Study | Nursing (baccalaureate nursing and Master of Nursing degrees, with other disciplines | 786 (210 BSN students) | Not related to inclusive pedagogy | The advanced interprofessional health care escape room provided a fun, interactive way to teach health professional students | Identified Student needs: within the healthcare workforce | The interprofessional health care escape room moves the focus from traditional hierarchical systems to an engaging and collaborative approach.  It provides an opportunity to experience the challenges and rewards that come with working toward a common goal in a diverse group. | Subcomponents of inclusive pedagogy not addressed | Subcomponents of inclusive pedagogy not addressed | Evaluation | None | None |
| INACSL Standards Committee, Rossler et al., 2021e:  USA | Grey Literature: Standards of Best Practice | N/A | N/A | N/A | Best practices for Sim-enhanced interprofessional education should develop the design in consultation with experts and representatives of the targeted learners.  They also should consider multiple experiences to achieve expected outcomes, and design the simulation to meet the needs of a diverse learner population. | Knowledge regarding simulation, design principles, pre and debriefing methods evaluation strategies; |  |  | Knowledge regarding simulation, design principles, pre and debriefing methods evaluation strategies; Jeffries Simulation framework; interprofessional education aligned theoretical frameworks is recommended. A needs needs-based assessment recommended. | N/A | N/A | N/A |
| INACSL Standards Committee, Bowler et al., 2021d:  USA | Grey Literature: Standards of Best Practice | N/A | N/A | N/A | All stakeholders involved in the simulation experience must have a safe psychological and physical learning environment.  All stakeholders are expected to be honest, mindful, and sensitive to all differences and ethical issues related to the simulation experience. | Practice inclusion by respecting equity, diversity, and inclusivity among all involved and in all aspects of the simulation. Be  cognisant of issues related to the care of diverse populations and the diversity among all involved in the simulation. Be aware of diverse worldviews and individual differences that characterise patients, populations, and the health team.  Be respectful of the unique perspectives related to cultures, values, roles, responsibilities, and expertise of other health professions. |  |  |  | N/A | N/A | N/A |
| INACSL Standards Committee, McDermott et al., 2021c:  USA | Grey Literature: Standards of Best Practice | N/A | N/A | N/A | Decrease learner anxiety and increase psychological safety by providing preparation for scenario content. If prepared, learners are likely to feel comfortable carrying out scenario requirements and discuss during debriefing.  Incorporate activities that help establish an environment of integrity, trust, and respect by Simulationists being accessible and approachable.  Prevent defensive behaviour and support risk taking which supports learning and development of a professional identity. | Preparation and pre-briefing pre-empt difficulties identifies student needs and promote psychological safety. | N/A | N/A | N/A | N/A | N/A | N/A |
| INACSL Standards Committee, Persico et al., 2021b:  USA | Grey Literature: Standards of Best Practice | N/A | N/A | N/A | To promote psychological safety, provide information/preparatory activities and practice time before the simulation.  Discuss ground rules; acknowledge mistakes may happen, discuss the fiction contract and hold a pre-brief before the simulation. | T&L strategies and student diversity are essential.  Facilitation methods may vary while considering cultural and individual differences that affect learners’ knowledge, skills, attitudes, and behaviours.  Effective facilitation requires a facilitator who has specific skills and knowledge in simulation that including having an awareness of how the diversity of participants and others involved in the simulation-based experience may impact the learning experience. | N/A | N/A | N/A | N/A | N/A | N/A |
| INACSL Standards Committee, Watts et al., 2021a:  USA | Grey Literature: Standards of Best Practice | N/A | N/A | N/A | All simulation-based experiences require purposeful and systematic yet flexible and cyclical planning.  A script for a scenario or case is developed for consistency and standardisation to increase scenario repeatability/reliability.  Incorporate evidence-based components of cultural diversity within the simulation design/scenarios.  Refer to the Health Care Simulationist Code of Ethics regarding the creation of a safe educational environment.  Establish a psychologically safe learning environment during the prebriefing.  Plan an evaluation process to determine the quality or effectiveness of the simulation and use evaluation data for continuous quality improvement. | Preparation and a needs assessment are essential to pre-empt difficulties, identifies student needs, and promote psychological safety. Facilitators have training in simulation approaches.  Facilitators should be aware of the learner’s diverse cultural differences, values and responsibilities and consider that during the simulation design.  Include feedback from participants, peer clinicians and educators, stakeholders, and simulation program faculty and staff in the evaluation process. |  |  | Perform a needs assessment to examine the knowledge skills, attitudes and/or behaviours of individuals etc. Use the results of the needs assessment to create relevant, innovative, and interactive simulations.  Plan a learner-centred facilitative approach driven by the objectives, learners’ knowledge and level of experience, and the expected outcomes. | N/A | N/A | N/A |
| Jiménez-Rodríguez et al., 2020: Spain | Empirical Study | Third-year undergraduate nursing degree students | 59 | A mixed method was employed to analyse participants’ satisfaction and perceptions of simulated nursing video consultations. An online conference was conducted to establish a safe psychological learning environment. | The video consultations created a psychologically safe learning environment. | Identified student needs; in response to the COVID-19 pandemic. | This simulation experience can create a safe psychological learning environment providing an environment of trust and mutual support among students.  The students did not lose their calm during any of the cases, and they perceived the errors as part of their training. The psychological safety of these environments affects learning. | The students experienced technical problems during video conferences. | More studies are needed in this area, to conﬁrm the students’ satisfaction with simulated nursing video consultations.  Recreating simulated video consultations, was a response to the needs of simulation-based education prompted by the COVID-19 pandemic. Simulated nursing video consultations could be considered as another choice and could also be extended to other contexts. | Perception | None | None |
| Johnsen et al., 2021: Norway | Empirical Study | Second and third-year nursing students | 26 | This study aimed to explore nursing students’ perceptions of using a blended simulation approach, including hands-on simulation with simulated patients and a video-based serious game, in preparation for home healthcare clinical placements. | Students felt less exposed and actively engaged in a different way when playing the Serious Game, allowing for reflection.  The observer was identified as another alternative to learning.  The students agreed that it was a safe learning environment.  Students could make mistakes in a challenging but safe environment.  The Serious Game was perceived to be easy to use and a more flexible type of learning.  Students wanted to include the functionality to view students’ own correct/incorrect answers, and to undo wrong choices. | Not addressed | The blended simulation approach offered students supplementary ways of being active observers.  The blended simulation approach provided them with different but complementary learning experiences and reflections.  Students appreciated the inclusion of RNs and a real patient as actors in the blended simulation approach, as they made the simulations more realistic. | A few technical glitches due to an unstable internet connection were reported but overall students reported that they liked playing the SG. | Group composition and size could influence the quality of the experience and learning outcomes, the facilitating teacher should intentionally mix the groups so that students with different levels of experience and knowledge could learn from each other and possibly may decrease the chance of exclusion of students. | Perception | Collaboration team | None |
| Kim et al., 2021a: Korea | Empirical Study | Second-year nursing students | 21 | To understand the meaning and nature of nursing students’ virtual experiences, using VR and blended learning during a perioperative patient simulation.  Application of this method as an alternative or supplemental educational methodology | The experience of being a patient helped the participants understand the patient’s position, their language, and the behaviour and patient needs.  The participants were able to observe and experience the surgical process from the perspective of a patient using VR.  The participants became keenly aware that the patient’s experience must be included in the nursing curriculum. | Not addressed | Undertaking the patient's role as a patient during practical education is a method of indirect experience of patient care and a useful experience for a nursing student.  Through feedback from the participants, it was revealed that patient experience is an essential element that must be acquired by practitioners before undertaking direct experience in the nursing field. |  | There is a need to carefully pursue the inclusion of patient experience in nursing students’ practical education curriculum.  Research is required to evaluate nursing students’ competency development for patient-centred care through the experience of being a patient. | Experience | None | None |
| Kim et al., 2021b: Korea | Empirical Study | Senior baccalaureate nursing students | 20 | To understand prelicensure nursing students’ perceptions and experiences of using virtual simulation as an alternative to clinical practice during the coronavirus 2019 pandemic in South Korea.  Rapid response to learner needs in response to the COVID-19 pandemic. | Challenges were identified including that it was delivered in a non-native language. The difficulty varied among students, some had to use translation services.  Participants noted some culture-speciﬁc issues in the virtual simulation program that affected their comprehension.  The virtual simulation offered a more comfortable space for making and learning from mistakes.  They reported that being afforded experiences regardless of gender was an advantage e.g. maternity care provided by a male student.  Participants responded that they could practice repeatedly, self-evaluate and strengthen their abilities using quizzes, a feedback log, and reﬂection. | Identified student needs; Rapid response to learner needs in response to the COVID-19 pandemic. | Students can encounter difﬁculties due to language barriers and unfamiliarity with virtual simulation although it facilitates learning. | Nurse educators must be aware of challenges that students may face and support students based on their needs.  Instructors should remember that ﬁrst-time users might require guided orientation and time to familiarise themselves with virtual simulation.  More support is required for nursing students with low proﬁciency in English, by providing detailed instruction related to language and cultural differences before and after the simulation. | This study recommends providing a system to support students who have insufﬁcient English proﬁciency to maximise their learning. | Experience | None | None |
| Lasater et al., 2019:  USA, New Zealand, Australia | Empirical Study | Prelicensure/pre-registration nursing students States (US). | 532 | To determine what background variables inﬂuence what students notice and how they interpret what they notice about patients. To identify some implications for pedagogical approaches that may foster clinical judgement development among diverse learners. | Background variables can impact nursing students’ clinical judgement.  Clinical judgement does not represent “A one size fits all” approach.  Age was significant in two of the six questions. | Identified student needs: impact of learner diversity on student learning | Responses may have been rooted in how early in the program participants were.  Nurse educators must be familiar with and use a range of pedagogical approaches to meet the needs of individual learners.  Learning to think like a nurse is a unique developmental process for each learner, based on their backgrounds, and does not develop in predictable patterns. | The three site coordinators who reviewed the videorecording and the survey questions before the study were not as culturally diverse as the participants.  Diﬀerences in language, terminology, and accents likely contributed to participants' uncertainty when responding to survey questions. Including participants from all three countries in the pilot study may have been beneficial. | Perhaps students at this stage of learning need more concrete direction. Programs must seek ways to socialise students into their professional roles, congruent with an individual country's scope of practice, demographics, and health priorities. | Evaluation | None | None |
| McNeill et al., 2012:  USA | Empirical Study | Faculty members, BSN nurses as clinical teaching assistants | Fifty-three faculty members; 14 CTAs | Not related to inclusive pedagogy | Elements of the Evidence and Experience Workshop include: 1. Facilitator of learning: Reflective thinking; debriefing; learning theories; working with diverse learners; strategies.  2. Foster cognitive, psychomotor, and affective development in diverse learners.  After debrieﬁng, students provide feedback | Notes learner diversity | Although no research reports describing online or distance programs were found, the National League for Nursing’s Simulation Innovation Resource Centre provides 11 self-paced courses in the components of planning, implementing, and evaluating simulation. | There is no consensus for evidence-based practice in faculty development for simulation, possibly because a one-size-fits-all approach does not effectively serve the diverse range of faculty needs. | Designing faculty development for simulation within a framework for systems change, maintaining flexibility to meet diverse needs, and using existing online resources. | None | Evaluation Educator focus | None |
| Musgrove, 2016:  USA | Empirical Study | Undergraduate second-year medical-surgical nursing students | 135 | Virtual patient simulation is a cost effective alternative to high-fidelity simulation. | Not addressed | Noted that a diverse sample was recruited. | Definition of diversity from (AACN, 1997)  The benefits of virtual simulation include: it can be delivered via the World Wide Web, can be available, repeatable, accessible by multiple students at multiple locations at any time. | Students did not like having to type their responses and this may have impacted their learning and satisfaction scores.  The software does not have a drop-down menu for the students to select an option and the software does not have voice-to-text built-in to type what the students state. |  | Satisfaction and exam scores: | None | None |
| Ozkara San, 2018:  USA | Empirical Study | Associate Degree in Nursing students | 69 | To examine the effect of the Diverse Standardized Patient Simulation cultural competence education strategy on students’ transcultural self-efficacy.  . | Diversity extends to the student population in terms of age, ethnicity, English as a second (other) language, immigration status, and previous healthcare experiences  The simulation increased students’ transcultural self-efficacy perceptions.  All students irrespective of background benefited from formalised cultural competence education. | Learner diversity on student self-efficacy of engaging in simulation | The scenarios can assist educators to incorporate various culture-specific and multiracial data representing different ethnic, gender, racial, gender, socioeconomic, age, and religious groups.  Such opportunities would create a culturally inclusive and realistic learning experience and support culturally diverse students’ cultural competence. | A convenience sample was used: a small sample size (n = 53), a predominantly Catholic White cohort, and no use of a control group | Repeat studies with larger, diverse samples in a variety of geographic locations.  Future researchers should continue to gather demographic data.  Learner-centred, carefully designed and validated teaching and learning strategies, guided by a theoretical framework and international guidelines and Standards are valuable guides in implementing cultural competence education. | Perception | None | None |
| Rim and Shin, 2021: Korea | Empirical Study | Undergraduate nursing students | 16 | Not related to inclusive pedagogy | For each scenario, the students could repeatedly practice the task and they then performed the test.  In mannequin simulations, the task can only be performed once, but in virtual reality, the students could engage independently.  The themes showed independent learning as students build on their knowledge through repeated and continuous analysis of the situation. | Not addressed | Learners felt that technical difficulties often restricted the sense of immersion, and preferred moving their avatars directly. | The students reported initial difficulties manipulating items and objects in the virtual simulation due to unfamiliarity.  Technical issues and learners' representation and interaction affect presence and immersion in the virtual simulation. | The technical level of the platform and virtual fidelity should be considered when designing virtual simulation. Authors recommend the provision of effective virtual simulations in nursing education, using the template outlined in this study. | Evaluation | Evaluation | Evaluation |
| Saab, et al., 2021: Ireland | Empirical Study | Third-year undergraduate nursing students | 26 | To explore nursing students' perspectives on incorporating virtual reality in nurse education | Participants believed that virtual reality is novel, fun, memorable, inclusive, and engaging. Many believed that virtual reality would complement current T&L approaches, help build confidence, and provide nursing students with a safe space for trial, error, and problem-solving. Participants identified challenges with the required resources needed to use the virtual simulation; they also recommended continuous feedback regarding this issue. | Identified student needs. Highlighted that virtual reality could accommodate diverse learning styles and provide individualised teaching and learning experiences. | Virtual reality can enhance learning through repeated exposure to content and related clinical skills.  The results indicated that ‘sensing’ and ‘visual’ were the preferred learning styles among nurses regardless of gender, age, or experience.  Virtual reality was perceived to promote equity among students, especially when exposure to certain clinical experiences is limited. | Sight problems, vertigo, dizziness, motion sickness, and risk for injury were perceived to limit the use of virtual reality.  Educators who are adopting virtual reality as a teaching and learning strategy must address issues such as technology costs as well as space and training in virtual reality use. | Participants recommended embedding virtual reality in nursing curricula. Participants cautioned against replacing teaching and learning approaches with virtual reality, instead using it as an additional/supplemental resource to consolidate learning. Educators ought to consider the value of using virtual reality across diverse nursing curricula. The VR educational experience could be adapted and delivered on a standard desktop to reduce inequity for individuals who experience motion sickness. | Perception | None | None |
| Saab, et al., 2022: Ireland | Empirical Study | Third-year undergraduate Nursing students | 26 | To explore nursing students’ views of using virtual reality in healthcare. | Participants perceived the virtual reality as enjoyable and inclusive.  Participants believed that the technology was more suitable for younger users.  The suitability of virtual reality for older adults was questioned, who might find it “scary” or “confusing” or experience “technophobia”  Minor technical difficulties were reported and recommended prior preparation in the use of the technology. | Recommended more research regarding the long-term effect of virtual reality interventions among more diverse participants. | Virtual reality is a promising novel approach to reaching potentially different groups of audiences. | People with a history of severe motion sickness should be instructed to use virtual reality with caution. | Recommended more mixed method and longitudinal research regarding the long-term effect of virtual reality interventions among more diverse participants.  Training, or a short demonstration, particularly for first-time users, is recommended to familiarise individuals prior to use. This would help users focus on the information being delivered and reduce noise caused by technical difficulties | Perception | None | None |
| Scott et al., 2021:  USA | Empirical Study | Prelicensure BSN students and faculty | 376 students; sixteen faculty members | To address the multiple personal stressors and altered environments that students were experiencing, faculty attempted to provide maximum flexibility in online meetings and examination times while still upholding standards. | Many students reported online group meetings were extremely helpful and encouraging.  Students found the public platform lecture recordings user-friendly, easy to access, and easily downloaded for listening offline.  Students reported the debriefing process allowed for open discussion and offered an opportunity for collaboration among novices and experts.  Instructors reported feeling connected despite the distance with students as more virtual meetings were offered. | Identified student needs, life needs, IT needs ; Rapid response to learner needs in response to COVID-19 pandemic | Students reported missing the hands-on clinical skills  Students were concerned that virtual simulations increased their workload. | Challenges reported by faculty and instructors included student limitations and time constraints.  Students reported that the online platform was often unstable and presented challenges in terms of audio and visual performance. | Recommendations include the standardising of the simulation evaluation process by using a structured survey. | Evaluation | Collaboration team | Collaboration team |
| Spalla, 2012:  USA | Empirical Study | first-year undergraduate nursing students | Eighty for quantitative survey; 2-5 for focus groups | To study the effect of connecting disparate groups of nursing students and faculty through web-conferencing to ascertain if there is an impact on cultural awareness, cultural competence and/ or self-efficacy. | Web-conferencing with diverse peers may help to increase the cultural competence of nursing students. Web-conferencing allows mass participation as it is not limited by cost, time away from home, time away from study, or time away from work. | Notes learner diversity; diverse patient population and “diverse nursing groups” | Cultural differences create opportunities to discover viable, affordable pedagogies to improve the cultural competence of nursing students that will improve the appropriateness and quality of nursing care to the entire population.  The Appreciation for the Role of Culture Model for Nursing Education toward Cultural Competency may be used to assist nursing faculty in planning concrete experiences with multicultural peers.  Blending cultural competence teaching strategies would help to meet the needs of students with different learning styles. | Participants were students from a Private Catholic College in Ohio  The sample size was small. | More research is warranted to include a larger, public university with a more diverse international student population.  A faculty champion could teach others regarding cultural competence pedagogies. Finding culturally diverse groups near campus for service-learning would provide students with the chance to learn from different cultures. Working with culturally diverse groups of students and their faculty as web-conferencing partners is another option. | Experience | None | None |
| Stanley et al., 2018:  USA | Empirical Study | Bachelor of Science in Nursing students | not specified | Not related to inclusive pedagogy | By becoming aware of cultural differences, the patients’ culture can be included in their care.  Students reported an awareness of the significance of cultural competence and includes incorporating cultural and religious preferences into nursing care. | Not addressed | Increased use of online delivery for nursing education necessitates course designs that promote student interaction and foster community. | The reported lack of interaction in most online learning environments coupled with the limited use of appropriate technologies are areas that nurse educators should consider as they design their online courses. | Nurse educators must consider pedagogies that support online learning. Students are more likely to be successful when they are engaged and active participants in their learning | Evaluation | None | None |
| Verkuyl et al., 2016: Canada | Empirical Study | Nursing students, nursing faculty/clinicians and two gaming experts. | Six students, 5 nurses, 2 experts | Not related to inclusive pedagogy | The students highlighted that the game could be used in situations where there were limited paediatric opportunities, it also helped transfer theory into a practice setting and helped them to prepare for exams. | Identified student needs | The usability test identifies design issues that will be used to develop the virtual serious game for health educators interested in using technology to promote learning.  Several design changes were recommended, based on the feedback. | Identified the need to test with male students and faculty.  Opportunity to act on the feedback from this study, make changes and conduct a further round of testing however, authors were constrained by time and budget. | Add visuals for content-heavy areas  More detailed information/prompts and instructions page.  Provide a rationale for correct and incorrect answers.  Clearer decision buttons and options.  Self-paced text do that the learner is in control.  Include a clock to indicate how much time has lapsed  Explanation regarding scoring.  Include a video bar. | Evaluation | Evaluation | Evaluation |
| Verkuyl et al., 2022: Canada | Empirical Study | Nursing students; and other healthcare faculty. | 568 players | To describe the evaluation process we used to collect data about the learner experience to design or improve virtual games that support student learning. | The simulations were positively received by a diverse group.  Some players reported that the options lacked clarity regarding response options which reduced autonomy, and inﬂuenced players’ sense of mastery or ability to make the decisions. | Educators need to have a better understanding of the user experience. The simulation was well received by a diverse group of respondents. | This study highlighted the importance of evaluation of user experience when designing and implementing a serious game.  Short, simple and effective tools are required as part of the ongoing development and improvement of serious games. | It is possible that the survey was largely completed by players who had a positive gaming experience.  Some technological issues were experienced. | Some suggestions to provide the option to fast-forward Players recommended that the choice descriptors for some of the decision points be improved.  Players suggested expanding the rationale for incorrect choices and adding rationale for correct choices. | Evaluation  Experience | Collaboration team | None |
| Verkuyl et al., 2018: Canada | Empirical Study | Nursing students and nursing faculty | 12-six students and six nursing faculty. | To describe the usability testing completed on a newly developed virtual gaming simulation for nursing students. | Occasionally, a participant did not like the options provided and wished they could make their own option. | Not addressed | Usability testing can be adapted to meet speciﬁc educational needs for testing virtual experiences.  The game enabled users to make mistakes, learn from them and repeat certain decision points; this feature was very important to millennial users. | Time and budget constraints prevented a repeated usability test once the adjustments were made. | The usability methodology can be used to test virtual experiences before a study or including virtual experiences into curricula. Staff and students would like the game and storyline to be longer.  All participants wanted more time with the client.  Some students wanted more detailed instructions at the beginning of the game. | Evaluation | Evaluation | Evaluation |
| Yehle, 2011:  USA | Empirical Study | Associate Degree Nursing Students | 200 | Not related to inclusive pedagogy | Students could choose to repeat scenarios benefiting all students. | Identified student needs | Virtual environments offer alternative simulation opportunities. Variation in student ability in using the platform was highlighted. Fatigue, job, school and family responsibilities were challenging for students. | Network connections caused issues throughout the study.  Platform instability | The use of artificial intelligence for communication.  Better bandwidth is required. Scenarios used can be levelled to suit the learner. | Experience | None | None |
| NLN, 2017:  USA | Grey Literature: Toolkit | N/A | N/A | N/A | As nursing students in the 21st century originate from increasingly diverse backgrounds, the demand for pedagogical approaches that are theoretically appropriate, equitable, inclusive, and responsive to diverse perspectives has grown.  Identifies questions for practicing inclusive pedagogies. | Identifies questions regarding recruitment and retention of diverse students. |  |  |  | N/A | N/A | N/A |
